# The Correlation Between Carbohydrate Loading Diet and Gut Microbiome: A Systematic Review

**DOI:** 10.1002/mbo3.70045

**Published:** 2025-08-05

**Authors:** Omar El‐Kholy, Lindsey Nichols, Ahmed Adham R. Elsayed, Marc D. Basson

**Affiliations:** ^1^ Faculty of Medicine Alexandria University Alexandria Alexandria Egypt; ^2^ College of Medicine, Northeast Ohio Medical University Rootstown Ohio USA; ^3^ Department of Surgery Northeast Ohio Medical University Rootstown Ohio USA; ^4^ Department of Anatomy and Neurobiology Northeast Ohio Medical University Rootstown Ohio USA

**Keywords:** Bacteroidetes, carbohydrate‐loading diet, Firmicutes, gut dysbiosis, gut microbiome

## Abstract

The gut microbiome critically influences digestion, mucosal permeability, metabolism, blood pressure, and lipid profile. Pathological shifts in these processes cause metabolic syndrome, a growing human health concern that may be modeled in animals with carbohydrate‐loading diets. We reviewed the effects of carbohydrate‐loading diets on the animal gut microbiome. A systematic literature search was performed up to September 2024 on five databases: Cochrane Library, PubMed, Scopus, Web of Science, and VHL. Relevant animal studies were assessed for risk of bias using SYRCLE's tool. Seventeen studies were included, with data from more than 690 rodents. Carbohydrate‐loading diets alter the gut microbiome composition, diversity, and ratios. High‐carbohydrate, high‐fat diets were almost consistently associated with an increased Firmicutes to Bacteroidetes (F/B) ratio. Different types of carbohydrates, such as fructose, sucrose, or even special diet types, vary widely in impacting both the microbiota and microbiota‐associated pathophysiology, inducing different metabolic states and affecting blood pressure, gut structural integrity, immunomodulation, and other functions. Interventions added with or after feeding substantially modulated these diet‐induced changes. Carbohydrate‐loading diets can differentially influence the gut microbiome and associated physiology. High‐fat carbohydrate diets, apart from starch‐based diets, typically increase the F/B ratio, a shift linked to human obesity. In contrast, low‐fat carbohydrate diets do not elevate the F/B ratio but instead produce diverse microbiome effects, ranging from beneficial to harmful, depending on the carbohydrate type and other influencing factors. Further animal and human research is crucial to validate and further illuminate the dietary impact on the gut microbiome.

## Graphical abstract

Carbohydrate‐loading diets alter the gut microbiome differently. High‐fat carbs raise the Firmicutes/Bacteroidetes (F/B) ratio, which is linked to obesity, while low‐fat carbs cause varied effects. Outcomes depend on carbohydrate type and other factors, highlighting the need for further research.

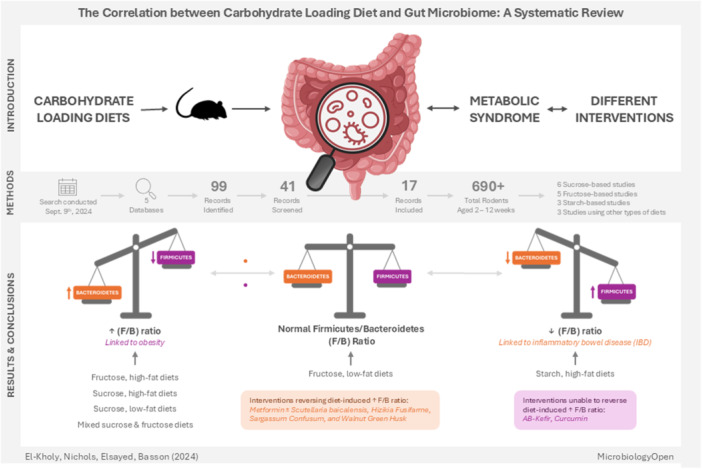

## 1 Introduction

**Figure 1:**
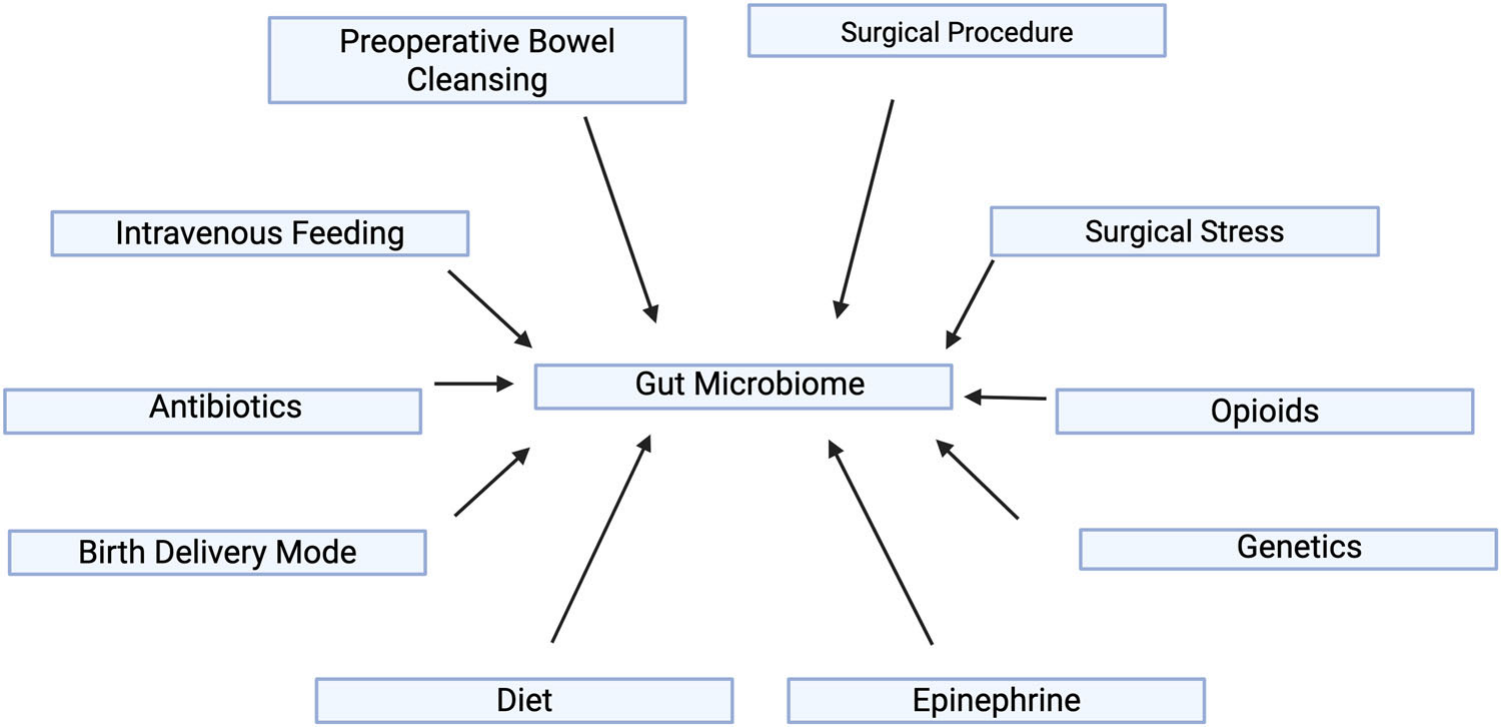
Influences on the gut microbiome. Created in BioRender. Nichols, L. (2025), https://BioRender.com/p67w278.

Diet during infancy and adulthood, antibiotic use, and birth delivery type are factors that influence the human gut microbiome (Jandhyala 2015; Yang et al. 2021) (Figure 1). In addition to these, surgical procedures may have a major impact on the gut microbiota (Stavrou and Kotzampassi 2017). The surgical procedure itself, coupled with other aspects of this procedure, such as preoperative bowel cleansing, vasoactive agents, opioids, surgical stress, and intravenous nutrition, can all lead to changes in the gut microbiome (Stavrou and Kotzampassi 2017) (Figure 1). Genetic predisposition and comorbidities determine the extent of the dysbiosis (Stavrou and Kotzampassi 2017; Fishbein et al. 2023; Y. Chen, Zhou, et al. 2021). Negative secondary outcomes and complications can arise from the dysbiosis caused by these interventions (Yang et al. 2021; Fishbein et al. 2023; Y. Chen, Zhou, et al. 2021). This is a severely understudied topic, so it is essential to review the articles available. Furthermore, the effect of a high‐carbohydrate diet on the human gut microbiome has not been studied. Although there are studies addressing the effects of foods high in digestible carbohydrates, such as dates, or non‐digestible carbohydrates, such as fiber, done in humans (Singh et al. 2017), they are addressing these foods as additives in the diet and not as a carbohydrate‐loading diet. Research on the effects of a carbohydrate‐loading diet on the gut microbiome has yet to be studied in humans. This systematic review aims to highlight the changes in the gut microbiome seen in rodents on carbohydrate‐loading diets, which can help more research to be done on this topic.

The human gut microbiome is dominated by two major phyla, Bacteroidetes and Firmicutes (Jandhyala 2015; Van Hul et al. 2024). Other abundant phyla include Verrucomicrobia, Pseudomonadota, and Actinobacteria (Van Hul et al. 2024). The colon microbiome has been the most well‐studied in health and disease states (Jandhyala 2015). Many different disease states are linked to changes in the Firmicutes/Bacteroidetes ratio (denoted as the F/B ratio) (Jandhyala 2015; Van Hul et al. 2024; Magne et al. 2020). Changes in the normal composition of the gut microbiome are called gut dysbiosis (Lee et al. 2024). This can impact many different bodily processes, such as the production of short‐chain fatty acids (SCFAs), which in turn have profound further effects on benign and malignant gut mucosal biology (Emenaker and Basson 1998; Basson et al. 1998; Morrison and Preston 2016). Furthermore, gut dysbiosis is linked to obesity, metabolic disorders like type 2 diabetes, and Parkinson's disease (Lee et al. 2024; Hamjane et al. 2024; Chiantera et al. 2023).

In general, humans and mice have similar bacteria at the genus level, but there are quantitative differences (Hugenholtz and de Vos 2017). Approximately 1500 species of bacteria have been identified in the human gut, but only 100 in the mouse (Hugenholtz and de Vos 2017). However, mice and humans also have a similar bacterial makeup at the phylum and family level (Kostic et al. 2013), with Bacteroidetes and Firmicutes also encompassing the most abundant bacteria in the mouse microbiome (Hugenholtz and de Vos 2017). Mice do have a larger abundance of Deferribacteres than humans (Hugenholtz and de Vos 2017). Thus, while there are differences between the two gut microbiomes, mouse models are currently one of the best options to translate to humans (Kostic et al. 2013; Hildebrand et al. 2013). Considering such studies, it should be noted that changes in the gut microbiota can be seen among different mouse strains, facilities, housing conditions, and dietary interventions (Hugenholtz and de Vos 2017; Hildebrand et al. 2013; Ericsson and Franklin 2021).

In both humans and rodents, diet critically influences the gut microbiota (Gomaa 2020). Differences in nutrient consumption alter the relative abundance of bacterial species (Gomaa 2020). In rodents, high‐fat diets may increase the F/B ratio, increase *Lactobacillus* and *Bifidobacterium*, and induce other changes in the relative abundance of the bacteria (Chae et al. 2024). High carbohydrate diets may also induce gut dysbiosis in rodents through an increase in the Proteobacteria phylum and Desulfovibrionaceae (Chae et al. 2024). However, more research is needed on this topic to further clarify the relationship between high carbohydrate diets and changes in the relative abundance of bacteria. In mice, a diet high in sucrose has been reported to cause insulin resistance and glucose intolerance and contribute to obesity (Y.‐T. Chen, Hsu, et al. 2021). Given the significant impact of diet on the gut microbiome, reversing gut dysbiosis caused by high‐carbohydrate diets may be a viable treatment option for obesity and other related complications (Y.‐T. Chen, Hsu, et al. 2021). Moreover, as fructose consumption continues to increase in the human diet (Hsu et al. 2020). It is essential to examine the effects of these changes on the gut microbiome and their potential consequences. During pregnancy, a diet high in fructose can lead to adverse outcomes in rodents, including hypertension for the mother and offspring (Hsu et al. 2020). Research should focus more on understanding how changes in the gut microbiome caused by high‐carbohydrate diets can contribute to various disease models in rodents. Such research can illuminate how high‐carbohydrate diets affect the human gut microbiome and human disease states. In addition, understanding interventions against the effects of a high‐carbohydrate diet in rodents might eventually translate to interventions in humans. Because the intake of carbohydrates is increasing in the US population (Gross et al. 2004) and globally (Clemente‐Suárez et al. 2022), which is also linked to an increase in metabolic disorders such as type 2 diabetes (Gross et al. 2004), understanding the effects of such carbohydrate loading and how they may be mitigated is becoming increasingly critical to human health.

## 2 Materials and Methods

### 2.1 Data Sources and Search Strategy

This systematic review followed the Preferred Reporting Items for Systematic Reviews and Meta‐Analyses (PRISMA) guideline (Page et al. 2021). The search was performed on the 9th of September 2024 using five electronic databases (Cochrane Library, PubMed, Scopus, Virtual Health Library (VHL), and Web of Science), from inception till September 2024, using the following search string: “Carbohydrate Loading” AND ((Gastr* OR Gut OR Intestin*) AND (Microbio* OR Flora OR Bact*)) (see Supplementary Table 1). After the extraction of all the articles from the mentioned databases, all the duplicates were removed. Two reviewers independently screened the titles and abstracts, then the full‐text articles for eligibility based on inclusion criteria and our research question (what is the correlation between carbohydrate loading diet and gut microbiome?). All studies assessed as ineligible from either stage were excluded. Disagreements were resolved by consensus between the two reviewers and, if necessary, consulted and settled by the senior authors.

### 2.2 Eligibility Criteria

We included all studies that were original animal‐model studies, with at least one group fed on any type of carbohydrate‐loading diet (not only homogenous carbohydrate diets), regardless of preparation methods, or any other feeding variables, with or without any other added intervention, and had the composition of the subjects' gut microbiome as an assessed outcome, regardless of the method or tool used for analysis.

We excluded any study that was not an animal‐model study, had no carbohydrate‐loading diet (whether a homogenous or a mixed diet), did not assess the outcomes right after the carbohydrate‐loading diet, did not assess the gut microbiota composition as an outcome, that were not in English, or was not an original study (reviews, meta‐analyses, etc.).

### 2.3 Data Extraction

All the data extracted was reviewed by a senior author, and any disputes were resolved through consensus between the co‐authors, and if necessary, through the senior authors.

### 2.4 Risk of Bias Assessment

All included studies were assessed for risk of bias using SYRCLE's risk of bias tool for animal studies (SYRCLE's risk of bias tool for animal studies 2024), which is an adapted version of Cochrane's risk of bias tool for clinical randomized trials (Higgins et al. 2011). The SYRCLE tool assesses selection, performance, detection, attrition, and reporting bias using 10 domains, namely: sequence generation, baseline characteristics, allocation concealment, random housing, blinding, random outcome assessment, blinding, incomplete outcome data, selective outcome reporting, in addition to any other sources of bias as its tenth domain. The tool contains signaling questions for each domain to help with the assessment process, to which each question is answered with Yes (low risk of bias), No (high risk of bias), or Unclear (unclear risk of bias). We decided that domains in which the signaling questions could not be clearly answered with Yes or No to be scored as Unclear. The two co‐authors independently assessed each article for risk of bias and study quality, and any discrepancies were resolved through discussion, and if necessary, through consulting the senior authors.

## 3 Results

### 3.1 Search

**Figure 2:**
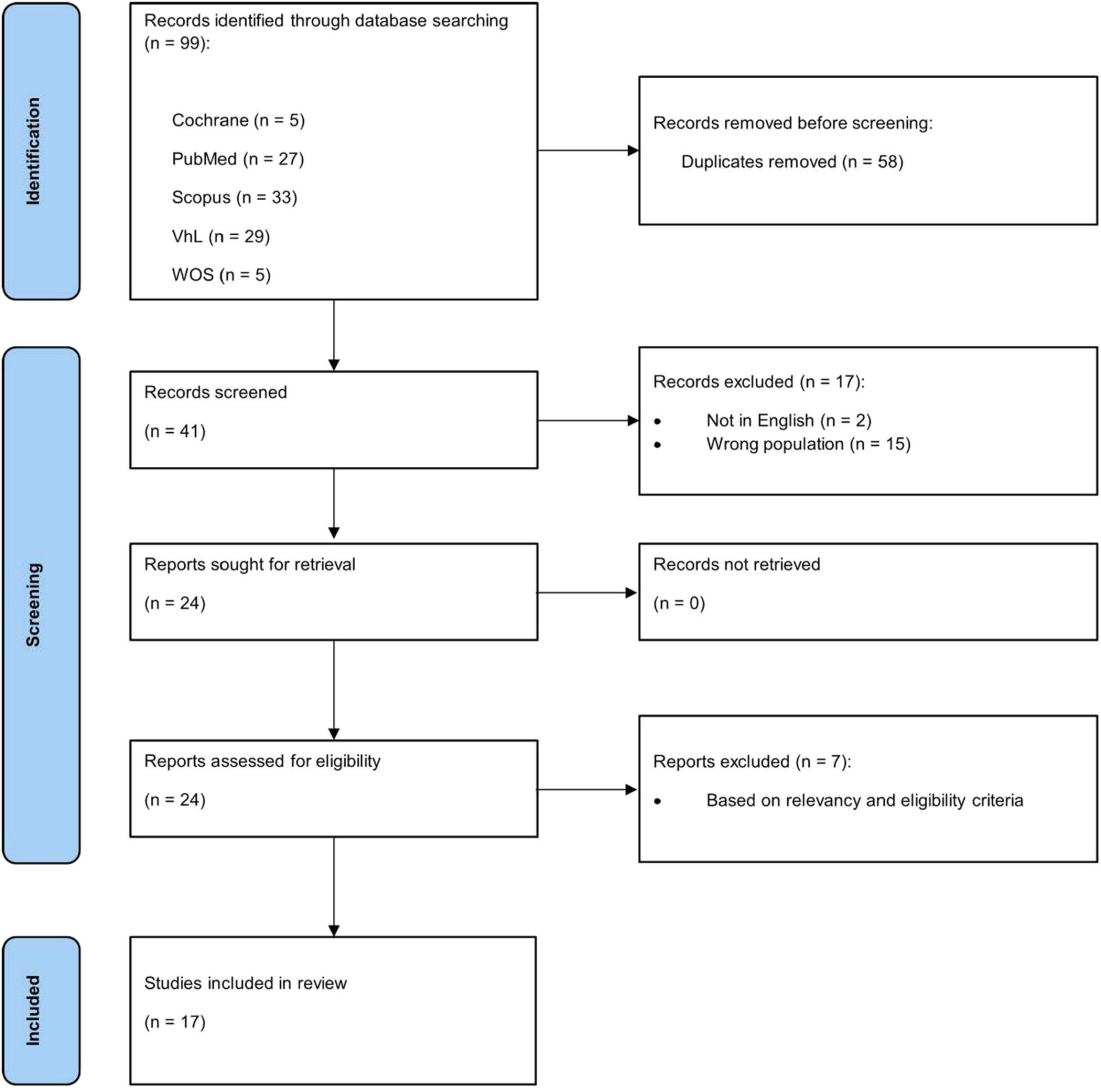
Preferred reporting items for systematic reviews and meta‐analysis (PRISMA) flow chart diagram.

The PRISMA flow diagram gives an overview of the selection process (Figure 2). The search process identified 99 possibly relevant articles, from which 58 were duplicates and were removed. Of the remaining 41 studies assessed for eligibility, 17 were excluded based on the title or abstract, and 7 were excluded after a full‐text analysis. A total of 17 studies were found to be eligible and were included in this review (Y.‐T. Chen, Hsu, et al. 2021; Hsu et al. 2020; Ansari et al. 2020; Asano et al. 2020; Barouei et al. 2017; Bidu et al. 2018; Bravard et al. 2021; Du Preez et al. 2019; Durand et al. 2020; Horne et al. 2020; Jia et al. 2020; Kong et al. 2019; Kumar et al. 2016; Milton‐Laskibar et al. 2021; Moreira Júnior et al. 2021; Wang et al. 2020; Yang et al. 2019).

### 3.2 Study Characteristics

Seventeen studies were included (Y.‐T. Chen, Hsu, et al. 2021; Hsu et al. 2020; Ansari et al. 2020; Asano et al. 2020; Barouei et al. 2017; Bidu et al. 2018; Bravard et al. 2021; Du Preez et al. 2019; Durand et al. 2020; Horne et al. 2020; Jia et al. 2020; Kong et al. 2019; Kumar et al. 2016; Milton‐Laskibar et al. 2021; Moreira Júnior et al. 2021; Wang et al. 2020; Yang et al. 2019), comprising over 690 subjects, with reported sample sizes varying from 20 (Barouei et al. 2017) to 120 (Du Preez et al. 2019) rodents. All the studies utilized rodents, and no non‐rodent studies were identified. All the studies used male rodents, except for one study (Kong et al. 2019) that used female mice. The reported ages of the rodents at acquisition were between 3 weeks (Ansari et al. 2020) and 12 weeks (Bravard et al. 2021). Different strains were represented: eight studies (Y.‐T. Chen, Hsu, et al. 2021; Ansari et al. 2020; Asano et al. 2020; Barouei et al. 2017; Bravard et al. 2021; Durand et al. 2020; Kong et al. 2019; Moreira Júnior et al. 2021) used C57BL/6 strains, one study (Bidu et al. 2018) used both Fat‐1 transgenic mice and wild‐type mice, and one (Wang et al. 2020) used Kunming mice. Three studies (Du Preez et al. 2019; Kumar et al. 2016; Milton‐Laskibar et al. 2021) used Wistar rats, and one (Hsu et al. 2020) used Sprague‐Dawley rats. One study (Jia et al. 2020) included both Kunming mice and Sprague‐Dawley rats, and finally, two studies (Horne et al. 2020; Yang et al. 2019) used Syrian Hamsters. Carbohydrate‐loading diets varied among the included studies; The main type of carbohydrate reported was sucrose in six studies (Y.‐T. Chen, Hsu, et al. 2021; Bidu et al. 2018; Bravard et al. 2021; Durand et al. 2020; Kong et al. 2019; Yang et al. 2019), fructose in five studies (Hsu et al. 2020; Ansari et al. 2020; Horne et al. 2020; Milton‐Laskibar et al. 2021; Moreira Júnior et al. 2021), one study (Du Preez et al. 2019) specified using a mixture of sucrose and fructose, and was varied, mixed, or unspecified in the remaining five (Asano et al. 2020; Barouei et al. 2017; Jia et al. 2020; Kumar et al. 2016; Moreira Júnior et al. 2021). Homogeneous carbohydrate diets were used in three studies (Y.‐T. Chen, Hsu, et al. 2021; Hsu et al. 2020; Wang et al. 2020), while the remaining 15 used a heterogeneous high‐carbohydrate high‐fat diet or other combinations. Four studies investigated the role of the diet only, while the remaining 13 had additional interventions investigated. Interventions included metformin (Ansari et al. 2020; Bravard et al. 2021); 3,3‐dimethyl‐1‐butanol (DMB) and 2,3,7,8‐tetracholorodibenzo‐*p*‐dioxin (TCDD) (Hsu et al. 2020), herring milt hydrolysate (HMH) (Durand et al. 2020); polysaccharides such as *Hizikia Fusifarme* (Jia et al. 2020), *Sargassum confusum* (Yang et al. 2019), *Scutellaria baicalensis* (Ansari et al. 2020), and Walnut Green Husk (Wang et al. 2020); probiotics (Kong et al. 2019); and other dietary supplements such as AB‐Kefir (Y.‐T. Chen, Hsu, et al. 2021), curcumin (Du Preez et al. 2019), inulin oligofructose (Kumar et al. 2016), omega‐3 polyunsaturated fatty acid (PUFA) (Bidu et al. 2018), and pterostilbene and resveratrol (Milton‐Laskibar et al. 2021) (Table 1).

**Table 1:** Study demographics and characteristics.

Author (date)	Animal model (species)	Gender and age	Number of animals	Type of carbohydrate loading diet	Intervention	Relevancy
Ansari et al. (2020)	Mice (C57BL6)	Male 3‐week‐old	49	High‐fructose, high‐fat	Metformin ± *Scutellaria baicalensis*	Partially relevant
Asano et al. (2020)	Mice (C57BL/6J)	Male 4‐week‐old	36	1975‐type Japanese Diet and Modern Japanese Diet	—	Partially relevant
Barouei et al. (2017)	Mice (C57BL6/J)	Male 6‐week‐old	20	High‐fat, high‐amylose maize (resistant starch) diet	—	Partially relevant
Bidu et al. (2018)	Mice (Fat‐1 Homozygous Transgenic and non‐transgenic littermate controls)	Male 12‐16‐week‐old	—	High‐fat, high‐sucrose	Omega‐3 polyunsaturated fatty acid (PUFA)	Highly relevant
Bravard et al. (2021)	Mice (C57BL/6J/Ola/Hsd)	Male 12‐week old	3 Groups (*n* = 5/6 animals per group)	High‐fat, high‐sucrose	Metformin	Highly relevant
Y.‐T. Chen, Hsu, et al. (2021)	Mice (C57BL/6)	Male 7‐week old	5 groups	High‐sucrose	AB‐Kefir	Highly relevant
Du Preez et al. (2019)	Rats (Wistar Rats)	Male 8–9‐week old	120	High‐carbohydrate (fructose and sucrose together), high‐fat	Curcumin	Highly relevant
Durand et al. (2020)	Mice (C57BL/6J)	Male 6‐week old	60	High‐fat, high‐sucrose	Herring milt hydrolysate	Partially relevant
Horne et al. (2020)	Hamsters (Syrian Hamsters)	Male ~100 g	27	Low‐fat, high‐fructose and high‐fat, high‐fructose	—	Highly relevant
Hsu et al. (2020)	Rats (Virgin Sprague‐Dawley Rats)	Male offspring	48	High‐fructose	3,3‐Dimethyl‐1‐butanol (DMB) 2,3,7,8‐tetracholorodibenzo‐*p*‐dioxin (TCDD)	Highly relevant
Jia et al. (2020)	Rats (Sprague‐Dawley Rats)	Male (rats ~120 g)	45	High‐sugar, high‐fat	Hizikia fusifame polysaccharide	Partially relevant
Kong et al. (2019)	Mice (C57 BL/6J)	Female 6‐week old	80	High‐sucrose (20% kcal protein, 70% kcal sucrose, and 10% kcal fat)	Probiotic (*Lactobacillus acidophilus, Bifidobacterium longum, Enterococcus faecalis*)	Highly relevant
Kumar et al. (2016)	Rats (Wistar Rats)	Male 8–9‐week old	48	Corn starch and high‐carbohydrate, high‐fat	Inulin oligofructose	Partially relevant
Milton‐Laskibar et al. (2021)	Rats (Wistar Rats)	Male 6‐week old	50	High‐fat, high fructose	Pterostilbene and resveratrol	Highly relevant
Moreira Júnior et al. (2021)	Mice (C57Bl/6)	Male 6‐week old	24	High‐fat (high‐sugar and butter)	—	Partially relevant
Wang et al. (2020)	Mice (Kunming Mice)	Male ~18–22 g	40	High‐fructose	Walnut green husk polysaccharide	Highly relevant
Yang et al. (2019)	Hamster (Syrian Golden Hamster)	Male 8‐week‐old	24	High‐fat, high‐sucrose	*Sargassum confusum*	Partially relevant

### 3.3 Summary of Outcomes

The 17 studies used different types of carbohydrate‐loading diets. The diets were either homogeneous carbohydrate‐loading diets (Y.‐T. Chen, Hsu, et al. 2021; Hsu et al. 2020; Wang et al. 2020), or in combination with other elements, predominantly an added high‐fat content. The types of carbohydrates also varied, such as fructose, sucrose, or other carbohydrates. These varieties in diet compositions certainly imposed a spectrum of results on the domains being investigated. This study focused on microbiome changes at the phylum level—primarily the two phyla linked with metabolic syndrome, Bacteroidetes and Firmicutes (Jandhyala 2015)—whether the findings reported changes in the F/B ratio, the relative abundances of the two phyla individually, or any genera belonging to either one of them. This study also examined the significant changes in other phyla and gut microbiome diversity and abundance findings, when reported.

Bacteroidetes and Firmicutes, along with the less prevalent Actinobacteria and Proteobacteria, dominate and represent the most diverse microorganisms in the adult gastrointestinal tract (Laterza et al. 2016). In addition, changes in Bacteroidetes and Firmicutes are often interdependent, reflected in the F/B ratio. Shifts in this ratio, whether positive or negative, are associated with various human diseases (Magne et al. 2020; Stojanov et al. 2020). Consequently, the abundance of Bacteroidetes and Firmicutes is commonly invoked in interpreting human microbiome data. This was therefore considered first. The relative abundance of Bacteroidetes was reported to increase in six studies (Y.‐T. Chen, Hsu, et al. 2021; Asano et al. 2020; Barouei et al. 2017; Horne et al. 2020; Kong et al. 2019; Wang et al. 2020) and decrease in four studies (Ansari et al. 2020; Bravard et al. 2021; Du Preez et al. 2019; Yang et al. 2019). In parallel, the relative abundance of Firmicutes increased in four studies (Ansari et al. 2020; Bravard et al. 2021; Du Preez et al. 2019; Yang et al. 2019), and decreased in three studies (Asano et al. 2020; Barouei et al. 2017; Wang et al. 2020). Despite these seemingly contradictory findings, two constant and interlinked patterns emerge. First, an inversely proportional pattern between the two phyla was observed. As Bacteroidetes increased, Firmicutes usually decreased, and vice versa, leading to corresponding positive or negative changes in the F/B ratio. Second, all high‐fat diets, apart from starch‐based diets, consistently produced an increase in the F/B ratio. This finding is consistent with obesity and dysbiosis in human studies (Magne et al. 2020). In contrast, low‐fat carbohydrate‐loading diets produced a spectrum of effects on the F/B ratio and other gut microbiome compositions. These effects varied according to the type of carbohydrate, animal model used, and other influencing factors. For example, all sucrose‐based diets, whether high‐fat or low‐fat, resulted in an increased F/B ratio. Meanwhile, low‐fat fructose‐based diets did not increase the F/B ratio. Details on specific changes in other types of gut microbiota in each study are presented (Table 2).

**Table 2:** Summary of outcomes.

Author (date)	Type of carbohydrate loading diet	Effect of carbohydrate loading diet on the gut microbiome	Intervention (if any)	Synergistic effect of carbohydrate loading diet with the Intervention	Impact on gut microbiome‐associated functions
Ansari et al. (2020)	High‐fructose, high‐fat diet (HFFD)	↓ Bacteroidetes ↑ Firmicutes	Metformin ± *Scutellaria baicalensis*	↑ Bacteroidetes ↓ Firmicutes	↑ Body weight, VAT weight, and liver color A lighter liver color indicates adipose tissue accumulation ↑ Glucose homeostasis, HbA1c level, combat fasting insulin, and insulin resistance (SB + MF)
Asano et al. (2020)	1975‐type Japanese Diet (JD) and modern Japanese diet (MD)	↑ Bacteroidetes ↓ Firmicutes (JD and MD)	—	—	↑ JD increased the occupancy rate of genera beneficial to the host, as in producing SCFAs, and a decrease in genera positively correlated with visceral fat accumulation.
Barouei et al. (2017)	High‐fat, high‐amylose maize (resistant starch) diet (HRS)	↑ Bacteroidetes ↓ Firmicutes	—	—	↑ Adiponectin and satiety hormones [RS‐HF] ↑ Immune signaling in the intestine [RS] Alteration of glucose and SCFA intestinal concentrations [RS] ↓ Intestinal, serum, and liver‐associated amino acids [RS]
Bidu et al. (2018)	High‐fat, high‐sucrose (HFHS)	[PHYLUM] ↓ Tenericutes in both fat‐1 and wild‐type (WT) mice ↓ Verrucomicrobia (fat‐1) disappear (WT) [GENUS] ↑ *Akkermansia* ↑ *Ruminococcus* (less in fat‐1 than WT) ↓ Clostridiales [FAMILY] ↓ S24‐7 in WT only	Omega‐3 Polyunsaturated fatty acid (PUFA)		Expression of transcripts for antimicrobial peptides was affected by the HFHS diet in WT mice when it not in fat‐1 mice.
Bravard et al. (2021)	High‐fat, high‐sucrose	↑ Firmicutes (*Clostridium*, Lachnospiraceae, Peptostreptococcaceae) ↓ Bacteroidetes (Muribaculaceae)	Metformin	↑ *Akkermansia muciniphila* ↑ *Adlercreutzia* ↑ *Propionibacterium* Reversal of HFHS diet changes	Prevention of metabolic disturbances [Short Metformin] Restoring the genetic expression in intestinal regions [Short Metformin] Modification of the bile acid pool. [Metformin]
Y.‐T. Chen, Hsu, et al. (2021)	Western Diet (WD; 40% kcal fat and 43% kcal carbohydrate “Sucrose”), High‐fat diet (HF; 60% kcal fat and 20% kcal carbohydrate)	↑ Overall diversity ↑ Bacteroidetes (*Parabacteroides*) ↑ Deferribacteres (*Mucispirillum*)	AB‐Kefir	Unable to reverse changes induced by the Western Diet	—
Du Preez et al. (2019)	High‐carbohydrate (fructose and sucrose together), High‐fat (H)	↓ Bacteroidetes ↑ Firmicutes disappearance of Actinobacteria	Curcumin	Unable to reverse changes induced by diet ↑ Clostridiales (Curcumin nanoparticles) ↓ Oscillopsia (Curcumin and curcumin nanoparticles)	Development of obesity, hypertension, dyslipidemia, and impaired glucose tolerance ↓ Blood pressure with higher doses of curcumin and lower doses of curcumin nanoparticles ↓ High‐carbohydrate, high‐fat diet‐induced fat deposition
Durand et al. (2020)	High‐fat, high‐sucrose	—	Herring milt hydrolysate (HMH1, HMH2, HMH3)	(HMH1) ↑ *Duosiella* Dec *Ruminoclostridum*, *Flavonifractor*, *Tyzzerella* (HMH2) ↑ *Lactobacillus* (HMH3) ↑ *Anaerotruncus* ↓ *Ruminoclostridum, Tyzzerella, Romboutsia*	Daily treatment with HMH2 and HMH3 improved early time point glycemia during the oral glucose tolerance test (OGTT) induced by the HFHS diet, without changes in weight gain and insulin secretion. Modulations of gene expression in the liver, such as the upregulation of sucrose nonfermenting AMPK‐related kinase (SNARK). HMH2 and HMH3 inhibited inducible nitric oxide synthase (iNOS) induction in J774 macrophages, but glucose uptake was not modified in L6 muscle cells.
Horne et al. (2020)	Low‐fat, high‐fructose (LFHF) and high‐fat, high‐fructose (HFHF)	(HFHF) Altered Firmicute/Bacteroidetes ratio (LFHF) ↑ Bacteroidetes (*Parabacteroides*, *Butryimonas*)	—	—	[HFHF vs. LFHF] ↑ energy intake ↑ epididymal white adipose tissue weight ↑ Liver weight to total body weight ratio Diet‐induced dyslipidemia
Hsu et al. (2020)	High‐fructose (HFR)	The main phyla were Bacteroidetes, Firmicutes, Verrucomicrobia, Proteobacteria, and Actinobacteria. (The Firmicutes to Bacteroidetes ratio was comparable between all groups)	3,3‐Dimethyl‐1‐butanol (DMB) 2,3,7,8‐tetracholorodibenzo‐*p*‐dioxin (TCDD)	DMB therapy affected several gut microbes related to TMA‐TMAO metabolism, including the phyla Firmicutes and Proteobacteria, families Enterobacteriaceae and Deferribacteraceae, and genus *Holdemania*.	[TCDD] ↑ HFR‐induced hypertension ↑ ADMA and SDMA ↓ l‐arginine‐to‐ADMA ratio ↑ AT1R and dec AT2R ↑ gut permeability [DMB] Attenuation of HFR‐induced hypertension Prevention of HFR and TCDD‐induced AT1R increase and AT2R decrease
Jia et al. (2020)	High‐sugar, high‐fat	↓ Firmicutes/Bacteroidetes ratio	Hizikia fusifarme polysaccharide (HFP)	Reversal of high‐sugar, high‐fat diet changes ↑ *Lactobacillus*, *Bifidobacterium*, *Ruminococcaceae*, *Alistipes*, *Turicibacter*, Lachnospiraceae_NK4A136_group, *Clostridium_sensu_stricto_1*, *Blautia*, *Faecalibaculum*, *Bacteroides*, *Roseburia*, *Romboutsia*, *Agathobacter*, *Weissella*, Oscillibacter, Eubacterium_coprostanoligenes_group, Eubacterium_xylanophilum_group, *Parasutterella*	HFP ↑ is the production of SCFAs HFP could positively affect inflammation, the intestinal barrier, and glucose metabolism disorders
Kong et al. (2019)	High‐sucrose (20% kcal protein, 70% kcal sucrose, and 10% kcal fat)	↓ *Lactobacillus*, *Bifidobacterium*, *Allobaculum*, *Alloprevotella*, *Clostridium sensu stricto*, *Faecalibacterium*, *Olsenella*, *Prevotella*, and *Ruminococcus* ↑ *Bacteroides*, *Alistipes*, *Acinetobacter*, *Anaerotruncus*, *Blautia*, *Dorea*, *Escherichia/Shigella*, *Oscillibacter*	Probiotic (*Lactobacillus acidophilus, Bifidobacterium longum, Enterococcus faecalis*)	↑ *Lactobacillus, Bifidobacterium, Lactococcus, Olsenella, Allobaculum, Clostridium sensu stricto* ↓ *Escherichia/Shigella, Oscillibacter, Acinetobacter*	Probiotic intervention can ↑ production of SCFAs, ↓ inflammation, ↑ glucose tolerance, and reverse metabolic disorders.
Kumar et al. (2016)	Corn starch and high‐carbohydrate, high‐fat	↑ Gut dysbiosis	Inulin oligofructose (IO)	↑ *Lactobacillus, Bifidobacterium*	IO increased gastrointestinal motility, improved gut‐barrier function, and may inhibit hepatic glucose production
Milton‐Laskibar et al. (2021)	High‐fat, high fructose	↓ Microbial alpha‐diversity ↓*Ruminococcaceae*	Pterostilbene and resveratrol	(Pterostilbene) ↑ *Akkermansia* and *Erysipelatoclostridium* ↓ *Clostridium sensu stricto 1*	Pterostilbene improved gut barrier function, intestinal integrity, and thought to be hepatoprotective
Moreira Júnior et al. (2021)	High‐fat (high‐sugar and butter)	↑ in *Blautia*, *Lachnoclostridium*, *Parvibacter*, *Bifidobacterium*, *Ruminoclostridum* ↓ *Lactobacillus*	—	—	↑ in pathogenic bacteria that damage the intestinal barrier leading to ↑ communication between the gut‐brain axis which influence feeding behavior through changes in gene transcription
Wang et al. (2020)	High‐fructose (HF)	↑ Bacteroidetes, Verrucomicrobia ↓ Firmicutes, Deferribacteres	Walnut green husk polysaccharide (WGHP)	Reversed the effects of the HF diet ↑ *Allobaculum*, *Blautia*, *Alloprevotella*	WGHP could help prevent chronic diseases caused by HF diet
Yang et al. (2019)	High‐fat, high‐sucrose	↑ Firmicutes, Proteobacteria, Actinobacteria ↓ Bacteroidetes	*Sargassum confusum* (SCO)	↓ Firmicutes/Bacteroidetes ↑ *Barnesiella, Coprobacter, Tannerella, Eubacterium, Clostridium XIVa, Lactobacillus* *↓ Allobaculum, Clostridium IV*	SCO could help prevent liver inflammation and type 2 diabetes

Abbreviations: ADMA, asymmetric dimethylarginine; AT1R, angiotensin type 1 receptor; SB + MF, *Scutellaria baicalensis* and Metformin; SCFA, short‐chain fatty‐acid; SDMA, symmetric dimethylarginine; TMA‐TMAO, trimethylamine‐trimethylamine oxide; VAT, visceral adipose tissue.

Thirteen of the included studies also considered additional interventions, such as metformin (Ansari et al. 2020; Bravard et al. 2021); DMB and TCDD (Hsu et al. 2020), HMH (Durand et al. 2020); polysaccharides (Ansari et al. 2020; Jia et al. 2020; Wang et al. 2020; Yang et al. 2019), probiotics (Kong et al. 2019); and other dietary supplements (Y.‐T. Chen, Hsu, et al. 2021; Bidu et al. 2018; Du Preez et al. 2019; Kumar et al. 2016; Milton‐Laskibar et al. 2021). The synergistic effects of these interventions with the carbohydrate‐loading diets greatly varied, whether on the gut microbiota or gut‐microbiota‐associated functions. The general aim in most of the studies was to use the proposed intervention to reverse the metabolic syndrome state induced by the carbohydrate‐loading diet. Many of the interventions reversed the gut microbiota changes induced by carbohydrate loading. These successful interventions included metformin (Ansari et al. 2020; Bravard et al. 2021), and the polysaccharides Hizikia fusifame polysaccharide (HFP) (Jia et al. 2020), *Sargassum confusum* (Yang et al. 2019), and Walnut Green Husk (Wang et al. 2020). These interventions also improved microbiome‐associated functions, reversing the inflammatory effects of the diets (Jia et al. 2020), suggested an ability to prevent chronic diseases (Wang et al. 2020) and helped prevent liver inflammation and type 2 diabetes (Yang et al. 2019), respectively. However, not all interventions were as successful. Neither AB‐Kefir nor curcumin was able to reverse diet‐induced changes to the microbiome (Table 2).

### 3.4 Risk of Bias

The SYRCLE's risk of bias tool for animal studies (2024) was used to evaluate the reviewed articles (Table 3). For Sequence Generation (#1); Ten studies (Y.‐T. Chen, Hsu, et al. 2021; Du Preez et al. 2019; Durand et al. 2020; Horne et al. 2020; Jia et al. 2020; Kumar et al. 2016; Milton‐Laskibar et al. 2021; Moreira Júnior et al. 2021; Wang et al. 2020; Yang et al. 2019) reported adequate sequence generation using randomization and were graded as “LOW” risk of bias; the remaining studies did not provide sufficient information and were graded as “UNCLEAR.” Baseline characteristics (#2) were clearly described in nine studies (Hsu et al. 2020; Asano et al. 2020; Barouei et al. 2017; Bidu et al. 2018; Du Preez et al. 2019; Durand et al. 2020; Horne et al. 2020; Kumar et al. 2016; Yang et al. 2019), earning a “LOW” risk of bias grade, with the remaining studies graded “UNCLEAR.” Neither allocation concealment (#3) nor random housing (#4) was reported in any of the reviewed studies, resulting in an “UNCLEAR” risk of bias grading. Similarly, none of the articles provided evidence of blinding procedures for caregivers or investigators, leading to “UNCLEAR” risk of bias grades for Performance Bias (#5) and Detection Bias (#7). One article (Du Preez et al. 2019) reported using random outcome assessment (#6) and was graded as “LOW,” whereas the remaining articles were graded as “UNCLEAR” due to insufficient information. Incomplete outcome data (#8) was addressed adequately in all studies, leading to a “LOW” risk of bias in this domain. Similarly, for the selective outcome reporting (#9) domain, all studies reported all outcomes adequately, scoring “LOW.” Finally, for the domain of other sources of bias (#10), we graded all articles as having an “UNCLEAR” risk of bias.

**Table 3:** SYRCLE's risk of bias tool.

Author (date)	1	2	3	4	5	6	7	8	9	Other
Ansari et al. (2020)	UNCLEAR	UNCLEAR	UNCLEAR	UNCLEAR	UNCLEAR	UNCLEAR	UNCLEAR	LOW	UNCLEAR	UNCLEAR
Asano et al. (2020)	UNCLEAR	LOW	UNCLEAR	UNCLEAR	UNCLEAR	UNCLEAR	UNCLEAR	LOW	LOW	UNCLEAR
Barouei et al. (2017)	UNCLEAR	LOW	UNCLEAR	UNCLEAR	UNCLEAR	UNCLEAR	UNCLEAR	LOW	LOW	UNCLEAR
Bidu et al. (2018)	UNCLEAR	LOW	UNCLEAR	UNCLEAR	UNCLEAR	UNCLEAR	UNCLEAR	LOW	LOW	UNCLEAR
Bravard et al. (2021)	UNCLEAR	UNCLEAR	UNCLEAR	UNCLEAR	UNCLEAR	UNCLEAR	UNCLEAR	LOW	UNCLEAR	UNCLEAR
Y.‐T. Chen, Hsu, et al. (2021)	LOW	UNCLEAR	UNCLEAR	UNCLEAR	UNCLEAR	UNCLEAR	UNCLEAR	LOW	LOW	UNCLEAR
Du Preez et al. (2019)	LOW	LOW	UNCLEAR	UNCLEAR	UNCLEAR	LOW	UNCLEAR	LOW	LOW	UNCLEAR
Durand et al. (2020)	LOW	LOW	UNCLEAR	UNCLEAR	UNCLEAR	UNCLEAR	UNCLEAR	LOW	LOW	UNCLEAR
Horne et al. (2020)	LOW	LOW	UNCLEAR	UNCLEAR	UNCLEAR	UNCLEAR	UNCLEAR	LOW	LOW	UNCLEAR
Hsu et al. (2020)	UNCLEAR	LOW	UNCLEAR	UNCLEAR	UNCLEAR	UNCLEAR	UNCLEAR	LOW	LOW	UNCLEAR
Jia et al. (2020)	LOW	UNCLEAR	UNCLEAR	UNCLEAR	UNCLEAR	UNCLEAR	UNCLEAR	LOW	LOW	UNCLEAR
Kong et al. (2019)	UNCLEAR	UNCLEAR	UNCLEAR	UNCLEAR	UNCLEAR	UNCLEAR	UNCLEAR	LOW	LOW	UNCLEAR
Kumar et al. (2016)	LOW	LOW	UNCLEAR	UNCLEAR	UNCLEAR	UNCLEAR	UNCLEAR	LOW	LOW	UNCLEAR
Milton‐Laskibar et al. (2021)	LOW	UNCLEAR	UNCLEAR	UNCLEAR	UNCLEAR	UNCLEAR	UNCLEAR	LOW	LOW	UNCLEAR
Moreira Júnior et al. (2021)	LOW	UNCLEAR	UNCLEAR	UNCLEAR	UNCLEAR	UNCLEAR	UNCLEAR	LOW	LOW	UNCLEAR
Wang et al. (2020)	LOW	UNCLEAR	UNCLEAR	UNCLEAR	UNCLEAR	UNCLEAR	UNCLEAR	LOW	LOW	UNCLEAR
Yang et al. (2019)	LOW	LOW	UNCLEAR	UNCLEAR	UNCLEAR	UNCLEAR	UNCLEAR	LOW	LOW	UNCLEAR

*Note:* 1: Sequence generation; 2: Baseline characteristics; 3: Allocation concealment; 4: Random housing; 5: Blinding (Caregivers and/or investigators, performance bias); 6: Random outcome assessment; 7: Blinding (Outcome assessor, detection bias); 8: Incomplete outcome data; 9: Selective outcome reporting; 10: Other sources of bias.

In summary, SYRCLE's risk of bias tool highlighted areas where the quality of the studies could be improved. Specifically, inadequate or unclear reporting was evident for sequence generation, allocation concealment, housing randomization, and blinding of caregivers and investigators. However, most studies ensured homogenous baseline characteristics and avoided confounding biases, and there was a low risk of bias for incomplete or selective outcome data. These findings highlight the importance of improved methodological reporting in future animal studies to enhance their reliability and validity (Table 3).

## 4 Discussion

### 4.1 Effects of High‐Carbohydrate Diets on the Gut Microbiome

The types of carbohydrate diets used in the 17 included studies varied, and the changes imposed on the gut microbiota were equally diverse. Whether the main carbohydrate type, the diet's homogeneity or heterogeneity (e.g., carbohydrate‐loading alone, high‐carbohydrate high‐fat diets, or additional dietary elements or designs), each approach presented different findings in the same domains. However, the constant finding in the gut microbiota analysis was the inverse proportion between the two dominant phyla, Bacteroidetes and Firmicutes. Where one increased, the other decreased, thus affecting the F/B ratio positively or negatively. Increased F/B ratio of the gut microbiota is widely linked with obese animals and humans compared with individuals of normal weight and is often considered a hallmark of obesity (Magne et al. 2020). However, it is important to note that multiple confounders, beyond dietary influences or metabolic state, can affect the F/B ratio. For instance, this ratio may vary across different rodent strains, human age groups, and even geographical regions (Magne et al. 2020). Alterations in the microbiome at the genus and species levels are also discussed, when possible, as these finer resolutions allow for more specific and insightful conclusions. While the effects of carbohydrate‐loading diets were diverse and heterogeneous, more careful analysis suggested some patterns that varied with the type of carbohydrate. These patterns are discussed in detail below.

#### 4.1.1 Effects of High‐Sucrose Diets on the Gut Microbiome

Sucrose‐based diets, the most commonly used type, were associated with an increased relative abundance of Firmicutes or related genera (Y.‐T. Chen, Hsu, et al. 2021; Bidu et al. 2018; Bravard et al. 2021; Kong et al. 2019; Yang et al. 2019) and a decreased abundance of Bacteroidetes or related genera (Kong et al. 2019; Yang et al. 2019), indicating a positive shift in the F/B ratio, a dysbiotic shift observed in obesogenic environments. This shift was observed in both high‐fat (Y.‐T. Chen, Hsu, et al. 2021; Bidu et al. 2018; Bravard et al. 2021; Durand et al. 2020; Yang et al. 2019) and low‐fat (Yang et al. 2019) sucrose‐based diets, highlighting the potentially pivotal role of sucrose itself, independent of dietary fat content, in shaping the gut microbiome. These results align with previous animal‐model research (Sun et al. 2021), which suggests that sucrose‐based diets can induce dysbiosis. This diet‐induced dysbiosis leads to the production of metabolites, such as SCFAs, that may contribute to the development of dyslipidemia and nonalcoholic fatty liver disease (Sun et al. 2021). This raises questions about the underlying mechanisms by which sucrose exerts these effects on the microbiome, potentially through altering fermentation processes, host energy metabolism, or systemic inflammatory pathways. Future research is needed to untangle these mechanisms and validate these findings, which were all observed in animal models, and to validate their relevance to humans.

At the family level, high‐fat sucrose‐based diets were characterized by the dominance of Tannerellellaceae (phylum Bacteroidetes) and Ruminococcaceae (phylum Firmicutes) (Y.‐T. Chen, Hsu, et al. 2021). Tannerllellaceae, notably decreased in abundance in ulcerative colitis human patients (Alam et al. 2020), highlights its potential relevance to gut health. Ruminococcaceae, on the other hand, includes beneficial genera such as *Ruminococcus bromii* and *Ruminococcoides bili*. These bacteria play a key role in fermenting resistant starches (RSs), indigestible to humans, into SCFAs like acetate, butyrate, and propionate. These SCFAs are vital for maintaining a healthy gut environment, modulating inflammation, and protecting against various illnesses (Kim et al. 2024).

At the genus level, high‐fat sucrose‐based diets appear to favor Akkermansia, with up to 60% of sequenced Firmicutes in one study corresponding to *Akkermansia muciniphila (*Bidu et al. 2018; Bravard et al. 2021). In contrast, low‐fat sucrose‐based diets were associated with a decrease in this genus (Kong et al. 2019). This raises the question of whether the differing fat content explains these contradictory findings on the beneficial bacterium or if other factors are at play. *A. muciniphila* is a commensal mucin‐degrading bacterium present in the human intestine from early life. Reduced abundance of *A. muciniphila* has been linked to various diseases in both humans and animal models, as it plays a pivotal role in maintaining a healthy gut barrier, regulating immunity, and limiting the onset of inflammation, a root cause of numerous diseases (Cani et al. 2022). However, recent studies have shown that an excessive abundance of *A. muciniphila* may be harmful rather than beneficial (Chiantera et al. 2023). Patients with multiple sclerosis and Parkinson's disease have an increased abundance of *A. muciniphila* (Chiantera et al. 2023). Additionally, Akkermansia supplementation in patients suffering from endocrine or gynecologic disorders may increase the risk of developing inflammatory bowel disease (IBD) (Chiantera et al. 2023). These findings underscore the necessity for future microbiome research to prioritize high‐resolution taxonomic analysis, particularly at the genus and species levels, rather than relying solely on broader phylum‐level classifications or simple taxonomic ratios. Such granular resolution is essential for uncovering functionally relevant microbial shifts and their mechanistic implications.

#### 4.1.2 Effects of High‐Fructose Diets on the Gut Microbiome

Fructose‐based diets, the second most common type studied, produced variable results, seemingly influenced by fat content (Hsu et al. 2020; Ansari et al. 2020; Horne et al. 2020; Milton‐Laskibar et al. 2021; Wang et al. 2020). Similar to sucrose, high‐fat fructose‐based diets significantly altered the composition of gut microbiota, increasing the F/B ratio (Wang et al. 2020). However, these changes were marked by shifts in the relative abundances of taxa already present, rather than by an increase in overall diversity or the introduction of new species (Ansari et al. 2020). In contrast, low‐fat fructose‐based diets had minimal or no effect on the F/B ratio (Ansari et al. 2020; Horne et al. 2020), with some even showing a decrease (Wang et al. 2020). At first glance, the disparity appears to stem from fat content, as the F/B ratio consistently increased with high‐fat diets, even aligning with evidence linking high‐fat intake to dysbiosis (Magne et al. 2020). However, additional factors, such as the animal models used, vary between hamsters (Ansari et al. 2020), mice (Ansari et al. 2020; Wang et al. 2020), and rats (Hsu et al. 2020; Horne et al. 2020), may also contribute to these differences. Notably, the decrease in the F/B ratio observed with low‐fat fructose‐based diets in Kunming mice (Wang et al. 2020) underscores the need to consider the species‐specific microbiome responses.

While high‐fat fructose‐based diets align with findings from high‐fat sucrose‐based diets, the divergent effects seen with low‐fat fructose diets suggest potential differences in how these carbohydrates influence the gut microbiome. This raises questions about whether sucrose and fructose affect distinct microbial or host pathways, be it fermentation, metabolic, or immunological.

In addition to the relative stability of the F/B ratio, low‐fat fructose‐based diets also resulted in a consistent increase in phylum Verrucomicrobia (Hsu et al. 2020; Wang et al. 2020). A notable species belonging to this phylum is *Akkermansia muciniphila*, a beneficial mucin‐degrading bacterium previously discussed in the context of sucrose‐based diets for its role in maintaining gut mucosal barrier integrity, which was increased with high‐fat sucrose‐based diets. However, recent research has suggested a link between an overabundance of *Akkermansia* and patients with Parkinson's disease and multiple sclerosis (Chiantera et al. 2023). Additionally, low‐fat fructose‐based diets enhanced the abundance of *Parabacteroides*, a genus linked with anti‐inflammatory properties (Ansari et al. 2020). These favorable outcomes suggest that fructose may be a promising candidate for human carbohydrate‐loading diets, whether used preoperatively or for athletic purposes, potentially avoiding the adverse microbiome changes observed with other carbohydrates. However, further research is essential to validate these findings in humans and determine their clinical relevance.

#### 4.1.3 Other Types of Carbohydrate‐Loading Diets and Their Impact on the Gut Microbiome

Starch, when used with high‐fat content, produced a consistent decrease in the F/B ratio (Barouei et al. 2017; Jia et al. 2020; Kumar et al. 2016). While this shift is still considered dysbiosis, a lower F/B ratio is more commonly associated with IBD rather than obesity (Stojanov et al. 2020). This contrasts sharply with the positive F/B ratio shifts observed in high‐fat sucrose‐ and fructose‐based diets, raising questions about the distinct mechanisms underlying these differences. In diets based on high‐amylose maize, classified as RS, the genus *Ruminococcus* was the most affected by the decrease in Firmicutes, though specific taxa like *Lactobacillales* and Erysipelotrichaceae were enriched (Barouei et al. 2017). Interestingly, *Ruminococcus*, which was increased in high‐fat sucrose diets, includes beneficial bacteria capable of fermenting RSs indigestible to humans into SCFAs. These findings highlight the complex interplay between dietary starches and the gut microbiota, warranting further investigation into the specific pathways driving these unique microbial responses.

When fructose and sucrose were combined in a single high‐fat diet (Du Preez et al. 2019), the gut microbiota exhibited an increased F/B ratio, with a rise in Firmicutes and a decline in Bacteroidetes, consistent with the effects of other high‐fat diets. Notably, this hybrid carbohydrate diet led to the disappearance of Actinobacteria, one of the four main phyla in the human gut, essential for maintaining the gut barrier, metabolic pathways, and immunological functions (Binda et al. 2018).

The 1975‐type Japanese diet (JD), rich in soy products, seafood, tubers, vegetables (including pickles), seaweed, mushrooms, and green tea, increased the relative abundance of Bacteroidetes, while decreasing Firmicutes, resulting in a lower F/B ratio compared to control diets composed of higher fat contents, simulating modern diets (Asano et al. 2020). This highlights the potential of traditional, plant‐based diets in promoting a microbial profile associated with improved gut health.

A high‐sugar, high‐fat diet using butter as the fat source produced distinct changes in the gut microbiota (Moreira Júnior et al. 2021). It increased the abundance of *Blautia*, *Lachnoclostridium*, and *Ruminoclostridium* within Firmicutes, along with *Parvibacter* and *Bifidobacterium* from Actinomycetota, while reducing *Lactobacillus* (Moreira Júnior et al. 2021). These findings emphasize how specific macronutrient compositions can uniquely reshape gut microbial communities.

### 4.2 Synergistic Effects of Carbohydrate Loading Diet and Interventions on the Gut Microbiome

#### 4.2.1 Metformin Intervention

**Figure 3:**
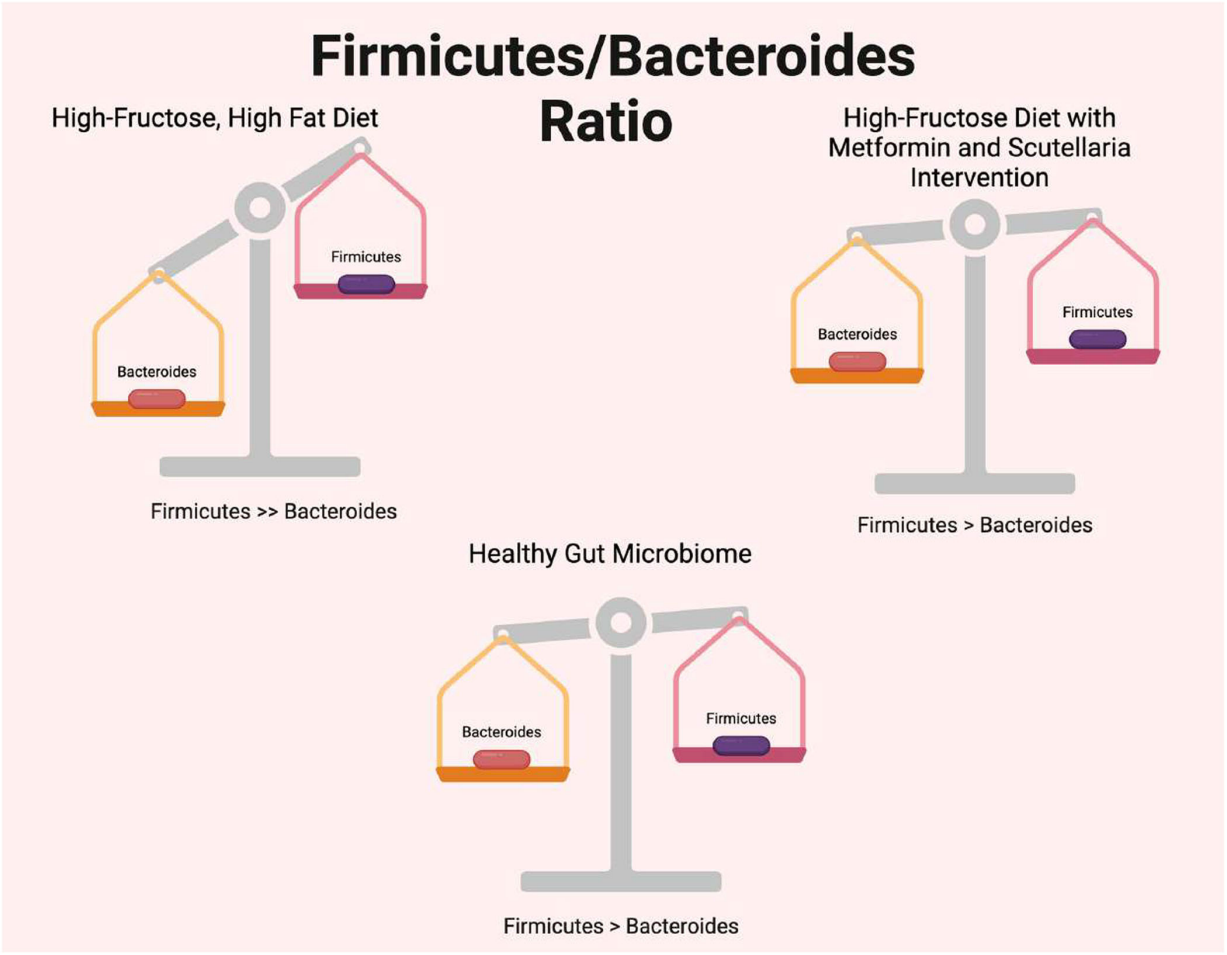
Comparing the effect of high fructose and fat diet with or without intervention on the Firmicutes/Bacteroides ratio to a healthy gut microbiome. Created in BioRender. Nichols, L. (2025), https://BioRender.com/q69s399.

Different types of high‐carbohydrate diets interact differently with metformin as an intervention (Ansari et al. 2020; Bravard et al. 2021). On a high‐fat, high‐fructose diet, metformin and Scutellaria (SB) restored the changes induced by the high‐fat, high‐fructose diet (Ansari et al. 2020). In particular, the intervention increased the relative abundance of Bacteroidetes while decreasing the relative abundance of Firmicutes (Ansari et al. 2020). This improved the F/B ratio (Ansari et al. 2020) (Figure 3). In comparison, metformin intervention on a high‐fat, high‐sucrose diet also reversed the changes in the gut microbiome induced by the diet (Bravard et al. 2021). However, there are differences in the gut microbiome bacterial changes between the high‐fat, high‐fructose, and high‐fat, high‐sucrose diets (Ansari et al. 2020; Bravard et al. 2021). Metformin intervention superimposed on the high‐fat, high‐sucrose diet led to an increase in *Akkermansia muciniphila*, *Adlercreutzia*, and *Propionibacterium* (Bravard et al. 2021). These are beneficial bacteria known to maintain the intestinal barrier, metabolize isoflavinoids to eqol, which is an antioxidant, and produce vitamin B12, respectively (Bravard et al. 2021). Indeed, metformin also decreased bacterial families associated with poor health outcomes like obesity and diabetes, such as Lachnospiraceae, Clostridiaceae, and Peptostreptococcaceae (Bravard et al. 2021). Overall, metformin had a positive effect on the high‐fat, high‐fructose, and high‐fat, high‐sucrose diets and reversed the gut microbiome changes induced by these diets (Ansari et al. 2020; Bravard et al. 2021).

#### 4.2.2 Prebiotic and Probiotic Interventions

While the F/B ratio offers a broad perspective on microbiome composition and its shifts, it only provides a rough estimate of the balance between pathogenic and beneficial bacteria. A more detailed analysis of the genus or species level alterations, or even quantifying microbial metabolites, can yield deeper, more specific insights into the effects of different dietary interventions. This concept is well‐illustrated by pharmacomicrobiomic interactions between prebiotics/probiotics and carbohydrate‐loading diets (Y.‐T. Chen, Hsu, et al. 2021; Kong et al. 2019; Kumar et al. 2016).

For example, probiotics were able to mitigate the negative effects of high‐fructose diets, markedly restoring the diversity of the microbiome and the abundance of beneficial bacteria, specifically *Lactobacillus*, *Clostridium sensu stricto*, *Prevotella*, *Alloprevotella*, and other butyrate‐producing bacteria. In addition, probiotics even increased the abundance of bacteria negatively associated with obesity, including *Akkermansia*, *Bifidobacterium*, and *Lactococcus*. Similarly, inulin oligofructose also markedly increased inflammatory biomarkers and improved the lipid profiles of rats that were fed a high‐carbohydrate, high‐fat diet (Kong et al. 2019; Kumar et al. 2016). Indigestible fibers, such as inulin, are fermented by the microbiota, and the latter then produces SCFAs as metabolites. High‐fiber diets and SCFAs are associated with a lower inflammatory state and a decreased risk of cardiovascular, renal, and endocrine disease, and a decreased risk of malignancy in humans (Guarner 2005; den Besten et al. 2013). On the other hand, AB‐kefir was only able to restore the microbiome balance and ameliorate inflammation in obese mice fed high‐fat diets, but not in mice fed a high‐carbohydrate diet (Y.‐T. Chen, Hsu, et al. 2021). The difference in effectiveness of these prebiotics and probiotics likely has to do with the different strains of bacteria, the number of probiotics administered, and the differences in the compositions of the carbohydrate diets.

However, it is crucial to recognize that, much like the F/B ratio's limitations, categorizing microbiota as strictly ‘beneficial’ or ‘pathogenic’ is context dependent. Even typically beneficial bacteria like *Akkermansia muciniphila* can exert pathogenic effects when overabundant, underscoring the complexity of microbial ecological balance (Chiantera et al. 2023).

#### 4.2.3 Marine Brown Algae Interventions

Several studies looked at the effects of marine brown algae on different high‐carbohydrate diets (Jia et al. 2020; Yang et al. 2019). The overall finding was that marine brown algae reversed the gut dysbiosis caused by high‐fat, high‐sucrose, and high‐sugar, high‐fat diets (Jia et al. 2020; Yang et al. 2019). Specifically, an increase in Bacteroidetes and *Lactobacillus* was seen when marine brown algae were used as an intervention in both of these diets (Jia et al. 2020; Yang et al. 2019). A decrease in Bacteroidetes is linked to obesity (Ley et al. 2006). *Lactobacillus* is beneficial through the production of butyric acid (Kong et al. 2019) and in the treatment and prevention of dyslipidemia (Jia et al. 2020). In addition to this change, a restoration in the F/B ratio was seen (Jia et al. 2020; Yang et al. 2019). Alterations in this ratio, as seen induced by the high‐carbohydrate diets, are associated with low‐grade inflammation (Yang et al. 2019). Furthermore, a high‐fat, high‐sucrose diet with the *Sargassum confusum* (SCO) intervention led to an increase in *Lactobacillus, Barnesiella, Coprobacter, Tannerella, Eubacterium*, and *Clostridium XIVa* (Yang et al. 2019). A more abundant amount of *Barnesiella* has been linked with healthy individuals when compared to those with colorectal cancer or irritable bowel syndrome (Liu et al. 2020). *Clostridium XIVa* is also a beneficial bacterium that produces SCFAs and promotes an anti‐inflammatory response (Yang et al. 2019). Finally, a decrease in *Allobaculum* and *Clostridium IV* was seen (Yang et al. 2019). *Allobaculum* has been shown to increase in high‐fat diets and is positively correlated with the expression of angiogenin‐like protein 4 (ANGPTL4) (Zheng et al. 2021). ANGPTL4 regulates fat deposition, and increased levels are related to obesity (Zheng et al. 2021). However, *Clostridium IV* is linked to beneficial functions such as the production of SCFAs and its role in anti‐inflammatory processes (Grenda et al. 2022). In addition to the increase in *Lactobacillus* and Bacteroidetes, the high‐sugar, high‐fat diet with HFP intervention showed an increase in Muribaculaceae, *Akkermansia*, *Bifidobacterium*, Lachnospiraceae_NK4A136_group, *Olsenella*, *Blautia*, and Ruminococcaceae_UCG‐014 while decreasing the abundance of *Escherichia‐Shigella* (Jia et al. 2020). *Akkermansia* and *Bifidobacteria* are known to have positive effects (Jia et al. 2020). *Akkermansia* improves intestinal integrity and reduces chronic inflammation (Jia et al. 2020; Milton‐Laskibar et al. 2021) while *Bifidobacteria* regulate intestinal pH, promote the growth of beneficial bacteria, and mitigate diabetes (Jia et al. 2020). The decrease in *Escherichia–Shigella*, a bacterium linked to inflammation, shows the positive effects the intervention had on the gut microbiome (Jia et al. 2020). The administration of marine brown algae reversed the changes in the gut microbiome induced by the high‐fat, high‐sucrose, and high‐sugar, high‐fat diets.

#### 4.2.4 Other Interventions

Walnut Green Husk Polysaccharide (WGHP) was able to reverse the changes induced by high fructose consumption (Wang et al. 2020). WGHP administration reversed the decreased F/B ratio from the high fructose diet (Wang et al. 2020). Additionally, WGHP intervention reduced the amount of Verrucomicrobia and increased the relative abundance of Deferribacteres (Wang et al. 2020). Verrucomicrobia is beneficial for metabolism, but the decreased abundance is thought to be caused by the increase in fiber due to the intervention (Wang et al. 2020). The increases in Deferibacteres, known for their effect in regulating the immune system, provide another positive outcome after WGHP intervention on the high‐fructose diet (Wang et al. 2020). Additionally, WGHP led to an increase in *Allobaculum*, *Blautia*, and *Alloprevotella* compared to the high‐fructose diet group (Wang et al. 2020). These bacteria are thought to produce SCFAs (Wang et al. 2020). Furthermore, the WGHP intervention decreased Muribaculaceae, *Lachnoclostridium*, and *Akkermansia*, all of which were increased on the high‐fructose diet (Wang et al. 2020). Since WGHP was effective in reversing the changes caused by the high‐fructose diet, including reversing the F/B ratio, it may be an effective intervention to combat the changes in the gut microbiome seen on a high‐fructose diet.

One study of pterostilbene suggested that it can dose‐dependently affect the changes caused by the high fructose diet (Milton‐Laskibar et al. 2021). In a low‐dose pterostilbene intervention (15 mg), there was an increase in *Akkermansia* and *Erysipelatoclostridium*, both of which are thought to be beneficial to gut health through improving gut barrier function and enhancing intestinal integrity, respectively (Milton‐Laskibar et al. 2021). Furthermore, there was a decrease in *Clostridium sensu stricto 1*, which is thought to be pathogenic through its link with NAFLD (Milton‐Laskibar et al. 2021). The higher dose of pterostilbene (30 mg) showed an increase in *Streptococcus* (Milton‐Laskibar et al. 2021). Increases in *Streptococcus* abundance are linked to atherosclerosis (Sayols‐Baixeras et al. 2023). This negative outcome is believed to be due to the dose–response profile of polyphenols (Milton‐Laskibar et al. 2021). A low dose is beneficial, and a high dose is a stressor (Milton‐Laskibar et al. 2021). Overall, these differences in gut microbiome composition due to differing amounts of pterostilbene are understudied. However, low doses of pterostilbene can reverse changes generated by a high‐fructose diet (Milton‐Laskibar et al. 2021). Further research is needed to understand the mechanism behind these changes and determine whether they can be translated to humans.

DMB intervention reversed the effect of TCDD and a high‐fructose diet on the gut microbiome (Hsu et al. 2020). At 3 weeks, a high‐fructose diet combined with TCDD showed an increase in the family Deferribacteraceae and genus *Holdemania*, but DMB intervention reversed these changes (Hsu et al. 2020). At 12 weeks, the high‐fructose and TCDD groups promoted the growth of the *Collinsella* genus, which is linked to atherosclerosis (Hsu et al. 2020). DMB intervention led to an increased amount of the genus *Butyrivibrio*, which is linked to reducing the risk of hypertension (Hsu et al. 2020). Overall, these changes to the gut microbiome suggest that DMB plays a protective role in restoring the gut dysbiosis caused by TCDD and a high‐fructose diet.

HMH, a fish by‐product made of lipids, minerals, proteins, and nucleotides, was studied to see its effectiveness in combating the gut microbiome changes associated with a high‐fat, high‐sucrose diet (Durand et al. 2020). Three different types of HMH were studied (Durand et al. 2020). The first was an HMH1, which consisted of only protein/peptide and nucleic acid (Durand et al. 2020). Overall, HMH1 treatment promoted the growth of *Dubosiella* and reduced the amount of *Ruminoclostridum*, *Flavonifractor*, and *Tyzzerella* compared to those on the high‐fat, high‐sucrose diet (Durand et al. 2020). Additionally, those administered the HMH2 intervention, containing protein/peptide, lipids, and nucleic acid, had an increased abundance of *Lactobacillus* compared to the high‐fat, high‐sucrose diet (Durand et al. 2020). Finally, HMH3 intervention, including protein/peptide, lipids, nucleic acid, and astaxanthin, led to increases in *Anaerotruncus* while decreasing *Ruminoclostridum*, *Tyzzerella*, and *Romboustia* (Durand et al. 2020).

Interventions that had limited effect on the changes induced by the high‐carbohydrate diets included curcumin and resveratrol (Du Preez et al. 2019; Milton‐Laskibar et al. 2021). This study found that curcumin, the major active ingredient in turmeric, was unable to reverse the increased abundance of Firmicutes and decreased abundance of Bacteroidetes caused by the high‐carbohydrate, high‐fat diet (Du Preez et al. 2019). Finally, resveratrol intervention on a high‐fat, high‐fructose diet did not exhibit relevant changes in restoring the gut microbiome (Milton‐Laskibar et al. 2021). This lack of reversal in the gut microbiome is hypothesized to be due to resveratrol's reliance on specific animal models and experimental conditions (Milton‐Laskibar et al. 2021). Thus, overall, metformin, prebiotics, probiotics, marine brown algae, WGHP, certain doses of pterostilbene, DMB, and certain types of HMH appear most promising for the potential reversal of the deleterious effects on the gut microbiome by high carbohydrate loading diets. In contrast, resveratrol and curcumin may be less promising.

### 4.3 Impact of Carbohydrate‐Loading Diets and Interventions on Gut Microbiome‐Associated Functions

In addition to its role in digestion, the gut microbiome plays other important roles, such as the maintenance of the structural integrity of the gut mucosal barrier, nutrient and drug metabolism, immunomodulation, and protection against pathogens (Jandhyala 2015). These functions can be affected by any changes to the gut microbiome's properties, which were evidently reported in the reviewed studies, whether this effect was induced by diet, intervention, or both. In general, carbohydrate‐loading diets, especially those with high‐fat content, are used in animal studies to induce a metabolic state similar to the human metabolic syndrome, with effects such as obesity, hypertension, dyslipidemia, and impaired glucose tolerance evident in many of the included studies (Hsu et al. 2020; Ansari et al. 2020; Du Preez et al. 2019; Durand et al. 2020; Horne et al. 2020; Kong et al. 2019; Kumar et al. 2016; Moreira Júnior et al. 2021).

#### 4.3.1 Impact of High‐Sucrose Carbohydrate‐Loading Diets on Gut Microbiome‐Associated Functions

In studies utilizing sucrose‐based carbohydrate‐loading diets (Y.‐T. Chen, Hsu, et al. 2021; Bidu et al. 2018; Bravard et al. 2021; Durand et al. 2020; Kong et al. 2019), the impact of the diets and interventions used varied. Sucrose‐based diets affected the expression of transcripts for antimicrobial peptides only in the wild type, but not the fat‐1 transgenic mice, a new mouse model for Omega‐3 research (Fleck et al. 2003). The interventions, when found, in these studies were all successful in the reversal of the negative impact of the diet on the gut microbiome; metformin prevented metabolic disturbances, restored the expression of important genes in intestinal regions, and modified the bile acid pool (Bidu et al. 2018; Bravard et al. 2021); Daily treatment with Herring‐Milt Hydrolysate (HHM2 and HHM3) improved early time point glycemia during the oral glucose tolerance test (OGTT) induced by the high‐fat high‐sucrose diet, without changes in weight gain and insulin secretion, as well as modulation of gene expression in the liver, such as upregulating the sucrose nonfermenting AMPK‐related kinase (SNARK), and inhibition of inducible nitric oxide synthase (iNOS) induction in J774 macrophages (Durand et al. 2020). Probiotics were successful in increasing the abundance of many beneficial bacteria, such as *Bifidobacteria*, which are known to decrease inflammation, improve glucose tolerance, and reduce gut leakiness. *Lactobacillus* and *Lactococcus* were also increased, in addition to butyrate‐producing bacteria, restoring the composition of the gut microbiome (Kong et al. 2019). Finally, *Sargassum confusum* was suggested to be able to prevent liver inflammation and type 2 diabetes, induced by high‐calorie diets (Yang et al. 2019).

#### 4.3.2 Impact of High‐Fructose Carbohydrate‐Loading Diets on Gut Microbiome‐Associated Functions

For studies utilizing fructose as their main carbohydrate, the impact varied as well. The combined treatment of Metformin and *Scutellaria baicalensis* (SB + MF) showed marked reduction in body, liver, and visceral adipose tissue (VAT) weight in the high‐fructose, high‐fat diet groups, with liver color also returned to normal, indicating attenuation of hepatic lipid accumulation and expression of lipid regulatory genes (Ansari et al. 2020). The combined SB + MF treatment also improved glucose homeostasis, HbA1c level, combated fasting insulin, and insulin resistance (Ansari et al. 2020). It also alleviated serum lipid profile, ameliorated hepatic enzymes, and improved adipose tissue status, intestinal integrity, and lipid accumulation (Ansari et al. 2020). The combined SB + MF treatment also showed the ability to regulate hypothalamic gene expression and protect against neuronal degradation (Ansari et al. 2020).

When comparing low‐fat and high‐fat fructose‐based diets in hamsters (Horne et al. 2020), no significant difference in body weight gain was observed, although the high‐fat diet resulted in an increased energy intake, a higher amount of epididymal white adipose tissue weight, and an increased ratio of liver weight to total body weight. Predictably, the high‐fat diet led to the development of dyslipidemia, but the low‐fat diet did not (Horne et al. 2020). Hamsters fed a high‐fat fructose‐based diet also expressed a decrease in fermentation pathways that control the production of the SCFA, propionate, which is linked to a reduction in weight gain and intra‐abdominal fat accretion in overweight adults (Horne et al. 2020). Negative correlations between the amino acids aspartate and asparagine biosynthesis and fasting metabolic parameters were found in the high‐fat group as well, in addition to an increase in production of isoleucine, an amino acid linked with inactivity, type 2 diabetes, and metabolic syndrome (Horne et al. 2020). In comparison, the gut microbiome of hamsters fed a low‐fat fructose‐based diet expressed an increase in abundance of *Parabacteroides*, a genus linked with anti‐inflammatory properties, an increased expression of the butyrate‐producing bacterium, *Butyricimonas*, which is linked to adipocyte regulation, and improved dyslipidemia (Horne et al. 2020).

High‐fructose (HFR) diet‐induced hypertension, which TCDD exacerbated (Hsu et al. 2020). This HFR‐induced and TCDD‐elevated hypertension was shown to be attenuated by DMB therapy during gestation and lactation (Hsu et al. 2020). The HFR and TCDD hypertension were associated with increased asymmetric dimethylarginine (ADMA) and symmetric dimethylarginine (SDMA) ‐two compounds that reduce the body's ability to produce nitric oxide, an important compound in maintaining a healthy endothelium (Fleck et al. 2003)‐ and a decreased l‐arginine‐to‐ADMA ratio, as well as an increased angiotensin 1 receptor (AT1R) and decreased angiotensin type 2 receptor (AT2R), which DMB prevented. TCDD‐induced elevation of blood pressure is combined with increased AT1R, activation of aryl hydrocarbon (AhR) signaling, and increased gut permeability (Hsu et al. 2020).

Resveratrol showed no significant impact on the gut microbiome in high‐fat fructose‐based diets. In contrast, pterostilbene had notable effects, increasing the abundance of *Akkermansia* and *Erysipelatoclostridium*, which are associated with improved gut barrier function and intestinal integrity, respectively (Milton‐Laskibar et al. 2021). Additionally, pterostilbene reduced *Clostridium sensu stricto 1*, a genus linked to nonalcoholic fatty liver disease NAFLD (Milton‐Laskibar et al. 2021). Suggesting a hepatoprotective role through microbiome modulation, a finding that requires further research in humans to assess its validation and relevance.

WGHP showed promise in countering the effects of high‐fructose diets, demonstrating a capacity to help prevent chronic diseases associated with such dietary patterns (Wang et al. 2020). These findings emphasize the role of bioactive compounds in improving gut and metabolic health.

#### 4.3.3 Impact of Other Carbohydrate‐Loading Diets on Gut Microbiome‐Associated Functions

High‐amylose maize, a RS, increased the production of adiponectin and satiety hormones without extensive modification to gene expression in adipose and liver tissues. RS also elevated immune signaling in the intestine and altered glucose and SCFA concentrations in the intestine. The increase in SCFAs induced by the addition of RSs to the diet aligns perfectly with prior research (Guarner 2005; den Besten et al. 2013). These starches undergo microbial fermentation, yielding beneficial metabolites such as butyrate and propionate, while simultaneously enriching bacterial taxa specialized in their breakdown. Notably, these SCFAs exhibit anti‐inflammatory properties and may reduce the risk of various disorders, such as inflammatory bowel diseases and certain malignancies (Guarner 2005; den Besten et al. 2013). These findings underscore the importance of moving beyond broad metrics such as the F/B ratio to investigate microbiome changes at finer resolutions (genus/species level) and analyzing the functional outcomes of their metabolites on the microbial ecosystem as a whole and correlating these findings clinically to possible health implications.

Intestinal, serum, and liver‐associated amino acids were reduced with RS consumption along with the high‐fat diet (Barouei et al. 2017); Hizikia Fusifarme Polysaccharide increased the production of SCFAs in the group that received it (HFP) (Jia et al. 2020). The HFP group also had a decrease in *Escherichia–Shigella*, which is thought to have a positive effect on inflammation, intestinal barrier injuries, and glucose metabolism disorders (Jia et al. 2020). In addition, the HFP group had an increase in *Akkermansia*, which is associated with reducing chronic inflammation (Jia et al. 2020). Muribaculaceae_norank and Mollicutes RF39‐noran*k* increased in the HFP group, and this increase is associated with diabetes symptom relief (Jia et al. 2020); Inulin oligofructose increased beneficial bacteria, which increased gastrointestinal motility, improved insulin sensitivity and glucose uptake, and decreased leaky gut and inflammation, which ultimately reduced the toxins entering the bloodstream (Kumar et al. 2016).

**Figure 4:**
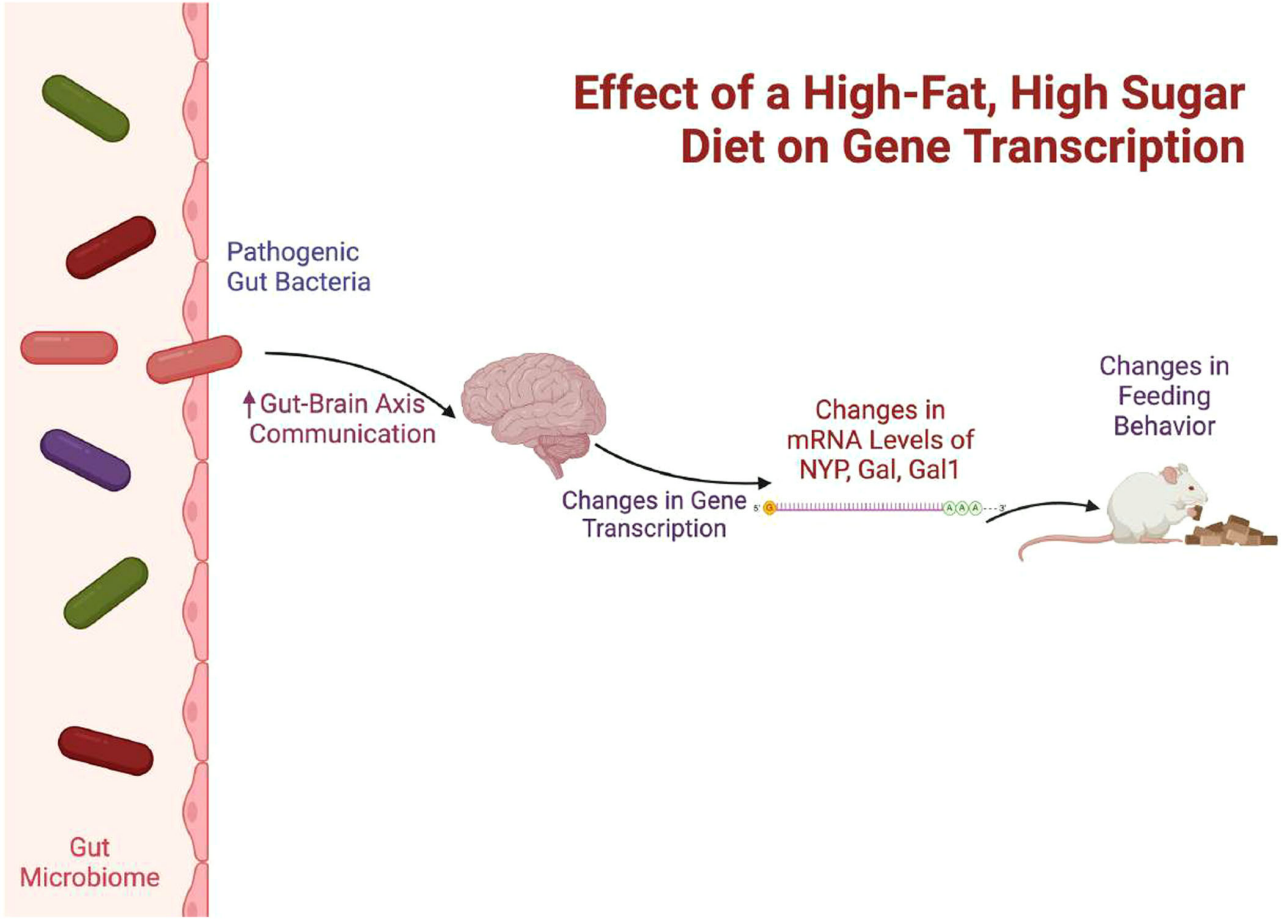
Effect of high sugar and fat diet on gene transcription leading to feeding behavioral changes. Created in BioRender. Nichols, L. (2025), https://BioRender.com/q44w612.

Rats fed on a diet of high‐fat, high‐carbohydrate (fructose and sucrose together) developed symptoms characteristic of human metabolic syndrome, as mentioned above. Curcumin was successful in reducing the blood pressure and hepatic fat deposition induced by this diet (Du Preez et al. 2019). However, it had no change in body weight or abdominal fat mass, nor did it reduce the basal blood glucose concentration (Du Preez et al. 2019). Mice fed on the 1975‐type JD had an increased occupancy rate of beneficial genera, such as those linked with producing SCFAs, and a decrease in genera positively correlated with visceral fat accumulation (Asano et al. 2020). A diet combining butter as the high‐fat component with high sugar increased pathogenic gut microbiota, damaging the intestinal barrier, and potentially facilitating communication along the gut‐brain axis (Moreira Júnior et al. 2021). This disruption influenced feeding behavior by altering the gene transcription of Npy, Gal, and Galr1 mRNA levels, underscoring the diet's role in modulating appetite and energy balance (Moreira Júnior et al. 2021) (Figure 4).

### 4.4 Type of Rodents as a Key Confounder in Gut Microbiome Alterations

Variety in rodent strains can be a confounding factor in the microbiome alterations, besides the dietary interventions (Magne et al. 2020). This is evident when comparing studies utilizing similar carbohydrate‐loading diets while using different models. For example, while a high‐fat, high‐fructose diet markedly reduced Firmicutes populations (particularly Ruminococcaceae) in Wistar rats, a similar diet left Firmicutes levels unchanged in C57BL/6 mice, though these mice showed significantly lower Bacteroidetes compared to controls (Ansari et al. 2020; Milton‐Laskibar et al. 2021).

Similar disparities emerge with high‐fat diets, high‐starch: C57BL/6 mice exhibited increased Bacteroidetes abundance, whereas Sprague‐Dawley rats showed decreased Bacteroidetes under comparable dietary conditions (Barouei et al. 2017; Jia et al. 2020). Notably, both studies reported decreased F/B ratios despite those opposing changes, indicating a difference in the magnitude of impact on the Firmicutes population between the two strains as well (Barouei et al. 2017; Jia et al. 2020). In addition, this highlights the critical limitation of relying solely on broad taxonomic ratios, emphasizing instead the need for finer‐resolution analyses and functional metabolic profiling to fully understand microbiome dynamics.

While this analysis attempts to highlight important intermodal differences, several caveats must be considered. While diets may be nominally similar, variations in feeding protocols (frequency, duration, amount), exact dietary composition, and housing conditions introduce additional confounding factors that may influence outcomes as significantly as diet itself. For instance, informal conversations with other researchers suggest that, in addition to the fact that different diets result in different rates of polyp formation in APC/MIN mice (Yu et al. 2001), the mice themselves spontaneously develop polyps or progress to carcinogenesis at different rates in different animal facilities for reasons that remain unclear (Yu et al. 2001).

Nevertheless, these discrepancies between rodent strains underscore the need for targeted research to better understand model‐specific microbiome responses. Furthermore, such findings emphasize the importance of correlating these observations with clinical confounders in humans, including age and ethnic variations, to improve translational relevance.

### 4.5 Limitations

This study may be limited by the retrospective collection of data, which could have led to study selection bias and results or data being misunderstood. Additionally, due to the numerous protocols included in each study, there may be inconsistencies in the results. Finally, it is crucial to highlight that this review mainly utilizes the F/B ratio as a marker of dysbiosis and attempts to discuss the alterations of bacterial taxa at a finer level (e.g., genus or species) whenever possible. While the F/B ratio provides a broad overview of microbiome alterations, it is important to highlight that the F/B ratio is not only influenced by dietary interventions, but also by other factors such as age, type of rodents, rodent strains, and even geographical regions (Magne et al. 2020).

## 5 Conclusion

The rodent and human gut microbiome have notable differences but seem to be the best alternative available currently (Hugenholtz and de Vos 2017). With the increased prevalence of high‐carbohydrate diets, the need to discover the effects of these diets is becoming more important than ever, and how to combat the changes they cause in the gut microbiome. This systematic review found that high‐carbohydrate diets, whether by themselves or mixed with another diet constituent such as a high‐fat component, alter the gut microbiome, and that this can lead to adverse outcomes such as changes in feeding behavior through gene transcription, impaired glucose tolerance, hypertension, and dyslipidemia (Hsu et al. 2020; Asano et al. 2020; Durand et al. 2020; Horne et al. 2020; Jia et al. 2020; Kumar et al. 2016; Milton‐Laskibar et al. 2021; Wang et al. 2020). However, certain interventions such as metformin, prebiotics, probiotics, and marine brown algae seem most promising for their ability to restore the relative relationship of the gut microbiome and are thought to be protective against the development of these outcomes. Although the effects of foods high in carbohydrates on the gut microbiome have been investigated (Singh et al. 2017), the effects of carbohydrate‐loading diets on the microbiome remain unexplored in humans; this area warrants systematic investigation. However, such studies must account for key confounders, including age, geographic location, and ethnic diversity, which are known to significantly influence microbial composition (Magne et al. 2020). These factors introduce variability even in animal models, where divergent outcomes have been observed across rodent strains under similar dietary conditions (Ansari et al. 2020; Barouei et al. 2017; Jia et al. 2020; Milton‐Laskibar et al. 2021). Moving forward, microbiome research should prioritize high‐resolution analyses (e.g., genus/species‐level profiling and metabolomics) over superficial assessments to yield more specific and insightful conclusions and correlate them clinically.

## Author Contributions

**Omar El‐Kholy:** conceptualization, writing – original draft, writing – review and editing, visualization. **Lindsey Nichols:** conceptualization, writing – original draft, writing – review and editing, visualization. **Ahmed Adham R. Elsayed:** conceptualization, writing – original draft, writing – review and editing, visualization. **Marc D. Basson:** conceptualization, writing – original draft, writing – review and editing, visualization, supervision.

## Ethics Statement

The authors have nothing to report.

## Conflicts of Interest

The authors declare no conflicts of interest.

## Supporting information


**Supporting Table 1:** Search strategy.

## Data Availability

Data sharing is not applicable to this article as no new data were created or analyzed in this study.
