# Supplementary material for: The Correlation Between Carbohydrate Loading Diet and Gut Microbiome: A Systematic Review
Source: Microbiologyopen. 2025 Aug 5;14(4):e70045. doi: 10.1002/mbo3.70045 (PMC12326083; doi:10.1002/mbo3.70045)
Supplement: Supplementary file 1 — Supporting Table 1: Search strategy. [file MBO3-14-e70045-s001.docx]

Supplementary Table 1. Search strategy

| **Search Strategy** | Cochrane Library  PubMed | "Carbohydrate Loading" AND ((Gastr* OR Gut OR Intestin*) AND (Microbio* OR Flora OR Bact*)) |
| --- | --- | --- |
|  | Scopus | "Carbohydrate Loading" AND ((“Gastr*” OR “Gut” OR “Intestin*”) AND (“Microbio*” OR “Flora” OR “Bact*”)) |
|  | Web of Science (WOS) | ["Carbohydrate Loading" AND ((Gastr* OR Gut OR Intestin*) AND (Microbio* OR Flora OR Bact*))](https://www.webofscience.com/wos/woscc/summary/18212791-e384-405c-907c-99b4e2e250d7-0133038750/relevance/1) |
|  | Virtual Health Library (VHL) | "Carbohydrate Loading" AND ((gastr* OR gut OR intestin*) AND (microbio* OR flora OR bact*)) AND instance:"regional" |
| **Date initiated** | 9/9/2024 | |
| **Initial Findings** | Total (n = 99)   - PubMed (n = 27) - Cochrane (n = 5) - WOS (n = 5) - Scopus (n = 33) - VHL (n = 29) | |
| **Removed Before Screening (Duplicates)** | Excluded (n = 58) (Duplicates)  Included (n = 41) | |
